# Muscarinic receptor regulation of chronic pain-induced atrial fibrillation

**DOI:** 10.3389/fcvm.2022.934906

**Published:** 2022-09-15

**Authors:** Chao Gong, Yu Ding, Feng Liang, Shuang Wu, Xiruo Tang, Hongzhang Ding, Wenjing Huang, Xiaotong Yu, Likun Zhou, Jun Li, Shaowen Liu

**Affiliations:** ^1^Shanghai General Hospital, Shanghai Jiao Tong University School of Medicine, Shanghai, China; ^2^Shanghai Jiao Tong University School of Medicine, Shanghai, China; ^3^Nanjing Medical University, Nanjing, China

**Keywords:** atrial fibrillation, chronic pain, autonomic nerve system, parasympathetic, M2 receptor

## Abstract

Atrial fibrillation (AF), one of the most common arrhythmias, is associated with chronic emotional disorder. Chronic pain represents a psychological instability condition related to cardiovascular diseases, but the mechanistic linkage connecting chronic pain to AF occurrence remains unknown. Wild-type C57BL/6J male mice were randomly divided into sham and chronic pain groups. Autonomic nerve remodeling was reflected by the increased atrial parasympathetic tension and muscarinic acetylcholine receptor M2 expression. AF susceptibility was assessed through transesophageal burst stimulation in combination with electrocardiogram recording and investigating AERP in Langendorff perfused hearts. Our results demonstrated the elevated protein expression of muscarinic acetylcholine receptor M2 in the atria of mice subjected to chronic pain stress. Moreover, chronic pain induced the increase of atrial PR interval, and atrial effective refractory periods as compared to the sham group, underlying the enhanced susceptibility of AF. Thus, autonomic cholinergic nerve may mediate mice AF in the setting of chronic pain.

## Introduction

Atrial fibrillation (AF) is one of the most common arrhythmia and cardiovascular diseases. It is tightly associated with high morbidity and mortality, due to secondary cardiac dysfunction, stroke, and myocardial infarction (1–3). Emerging studies have revealed that psychological stress and negative emotions, including depression (4, 5), stress (6, 7), and posttraumatic stress disorder (PTSD) (8), contribute to AF initiation and maintenance.

Multiple pieces of evidence indicate that chronic pain is associated with abnormal psychological stresses (9–11), and patients with chronic pain presented ECG changes including AF (12). In fact, acute or chronic pain is associated with an increased risk of cardiovascular disease (13–15). A systematic review demonstrated that chronic pain was significantly associated with mortality caused by cardiovascular disorders and implicated a possible dose–response relationship (16). The incidence of AF increases with age and is consistent with the age distribution of chronic pain.

The autonomic disorder is critical for arrhythmogenesis. In the atria, activity of the parasympathetic system enhances the G-protein-gated potassium channel (I_k−Ach_) to shorten the action potential duration (APD) and increases the spatial dispersion of refractoriness, facilitating ectopic excitation points and reentry (17). Cholinergic and adrenergic important autonomic nervous system on myocardium activities through G protein receptors expressed on the cardiac myocytes (18–21). Chronic pain induces cardiovascular dysfunction but the mechanistic linkage connecting chronic pain to AF occurrence remains unknown. Chronic pain caused autonomic nervous disturbances (22) and psychological-emotional disorder. There have been no reports regarding the effects of chronic pain on AF and the related mechanisms. Herein, we hypothesized that chronic pain might enhance the occurrence of AF by affecting the balance of the autonomic nervous system activity and signaling.

## Methods

### Animals and study groups

All the animal experimental procedures including feeding, intervention, and sampling followed the regulations of the Guide for the Care and Use of Laboratory Animals (NIH, No. 85–23) and were ethically authorized by the Nanjing Medical University Animal Ethics Committee (ethical code: IACUC-1808001). Wild type 8-week-old male mice, weighing 21–25 g on a C57BL/6J background, purchased from Nanjing Medical University Experimental Animal Center, were randomly divided into two groups: sham-operated (sham group, *n* = 10), and right sciatic nerve constricted (pain group, *n* = 14). All were maintained at a constant temperature (23–25°C), constant humidity (50–65%), and 12-h light or dark cycle with free access to general diet and water. All the mice were presented to the measurements detailed below.

### The chronic pain animal model and measurement of nociceptive behaviors

Pain-associated depression and stress are co-morbidities of chronic pain (23), which can be modeled in mice by chronic sciatic nerve compression injury (CCI) (24). To establish the chronic pain experimental model, CCI was performed on the right sciatic nerve, exposed at the mid-thigh level and constricted with four chromic gut (4/0) loose ligatures spaced at about 1 mm, according to a previously described method (24, 25). In the sham group, the right sciatic nerve was exposed without ligation. Then, 1 day before surgery and for 4 weeks afterward, punctate hyperalgesia was measured by the von Frey hair test on the right paw. The significant increase in the percentage of paw withdrawal frequency was regarded as a successful model of chronic pain. After surgery, mice were kept at 37°C until consciousness, and chow and water were guaranteed available *ad libitum*. Weighted every 5 days, mice were sure to have been given a normal diet and did not suffer from malnutrition and dehydration.

### Preoperative preparation

Then, 4 weeks after the successful establishment of the chronic pain and sham group mice, they were removed from their chambers and were anesthetized with an intraperitoneal injection of the sedative pentobarbital (20 mg/kg, Merck) and the analgesic fentanyl (0.125 mg/kg, Yichangrenfu Technology Co), and the anesthesia was considered successful by no movement of the clamped limb. Each was fixed in a supine position on the operating table, and a heating blanket was used to maintain body temperature in the normal physiological range (36.5–38°C).

### Electrocardiogram recordings

The electrocardiogram (ECG) was recorded continuously for 5 min *via* standard ECG limb lead II using four 25-gauge subcutaneous platinum electrodes (Grass Instrument, Quincy, MA) placed at the base of each limb. ECG signals were sampled three times by the system (BL-4205, Chengdu, China) after anesthesia, tracheal intubation, and artificial ventilation (SAR-1000, Yuyan, China; tidal volume = 6 ml/kg; respiratory rate = 110 breaths/min). Basic ECG parameters were measured, after artificial ventilation ECG stable, as follows: PR interval (the earliest P-wave onset to the earliest onset of the QRS complex onset), QT interval (the earliest Q or R-wave onset to the end of T wave), and HR.

### Investigation of susceptibility to atrial fibrillation

After collecting the basic ECG data, and supported by artificial ventilation through endotracheal intubation (tidal volume = 6 ml/kg; respiratory rate = 110 breaths/min), a 2-French catheter electrode (Japan Lifeline) was implanted in the esophagus dorsal to the left atrium through the mouth. The surface lead-II ECG was then recorded simultaneously. Transesophageal burst pacing (TEB) was implemented to investigate the susceptibility to AF using an automated stimulator (BL-4205, Chengdu, China). A 2-s burst pacing protocol was used (the first stimulation with a 40-ms cycle length, reducing by 2 ms in each successive burst to a 20-ms cycle length). A rapid irregular atrial rhythm, with disordered R-R intervals lasting at least 2 s, was defined as AF. The duration was gauged from the onset of the rapid irregular atrial rhythm triggered by TEB to the onset of the first normal sinus beat (26, 27).

### Investigation of the atrial effective refractory period

#### Establishing the langendorff-perfused assay

The Langendorff perfusion assay model is described elsewhere (28). It is a stable technique for studying electrophysiology characteristics. The whole heart is perfused with an appropriate perfusion solution, reducing the interference from central neural influences and from endogenous substances. After measuring basic electrophysiological parameters and maintaining anesthesia, opening the chest, and exposing the heart, the heart was rapidly excised and submerged in ice-cold Krebs-Henseleit (K-H) solution (NaCl 119 mM, NaHCO_3_ 25 mM, KCl 4 mM, KH_2_PO_4_ 1.2 mM, MgCl_2_ 1 mM, CaCl_2_ 1.8 mM, glucose 10 mM, and sodium pyruvate 2 mM, pH 7.4) bubbled with 95% O_2_ and 5% CO_2_. Aortic cannulation was performed using a tailor-made 21-gauge cannula prefilled with ice-cold K-H buffer placed outside the aortic valve. Then, the heart was quickly transferred and attached to the Langendorff perfusion apparatus (Radnoti, BIOPRBE), by which the aorta was continuously perfused with K-H solution, filtered by 5-μm filters, and warmed to 37°C by a water jacket and circulator, at a rate of 2.5 ml/min. After continuous stable perfusion for 30 min to reduce the effects of endogenous substances and drugs, the hearts with a pink color, which were spontaneously contracting, were further studied for their electrophysiological characteristics.

#### Investigating the left atrial effective refractory period

Paired platinum electrodes (1-mm inter-pole distance) were attached to the high left atrial epicardium (29) for programmed electrical stimulation (PES). PES was implemented by eight trains of burst pacing as S1 stimulation (8 Hz, 125 ms basic cycle length, 2 ms duration square-wave at three times the atrial excitation threshold), followed by a premature S2 extra-stimulation with a decreasing S1S2 interval. The S1S2 intervals from basic cycle length were successively reduced by 1 ms, every train depending on the atrial effective refractory period appearance. Bipolar electrogram (BEG) was collected from the high left atrial epicardium by a paired platinum electrode (1-mm inter-pole distance), and atrial effective refractory period (AERP) could be manifested as the longest S1S2 interval with a failed atrial signal initiated by S2 extra-stimulation (30).

### Investigating the role of the autonomic nervous system in atrial fibrillation

#### Parasympathetic effects in AF

After baseline electrophysiological experiments as above, intraperitoneal injection of the nonselective muscarinic receptor agonist carbachol (0.05 mg/kg, sigma, LRAB3674) over 2 min, TEB was implemented according to the preceding scheme to investigate AF susceptibility. Then, after 5 min of sinus recovery, the muscarinic receptor blocker atropine (0.5 mg/kg, Xinxiangshi Changle Pharmaceutical Co) followed by carbachol (0.05 mg/kg, sigma, LRAB3674) was administered by intraperitoneal injection, over 2 min, and TEB was implemented following the same procedure to investigate AF susceptibility. After a 5-min observation, the samples were collected.

#### Sympathetic effects in AF

After baseline electrophysiological experiments as above, intraperitoneal injection of the noradrenergic β receptor agonist isoproterenol (0.25 mg/kg, shanghai Harvest Pharmaceutical Co) over 2 min, TEB was implemented according to the preceding scheme to investigate AF susceptibility. Then, after keeping the sinus stable for 5 min, the β-receptor blocker propranolol (1 mg/kg, Sigma, BCBQ4523V) followed by isoproterenol (0.25 mg/kg, shanghai Harvest Pharmaceutical Co) was administered by intraperitoneal injection, over 2 min, and TEB was implemented following the same procedure to investigate AF susceptibility. After a continuous 5-min observation, the samples were collected.

### Western blot analysis

Western blot analysis was applied to quantitatively evaluate the expression of TH, Chat, β1, and M2. The frozen isolated atrial tissues were pulverized, homogenized on ice, and centrifuged at 14,000 *g* (4°C, 10 min) in a buffer. Protein concentration in the samples was assessed with the BCA protein assay (Beyotime), and 10 μg of protein was loaded on each lane. The samples were analyzed using 10% sodium dodecyl sulfate-polyacrylamide gel electrophoresis (SDS-PAGE) and transferred on polyvinylidene fluoride membranes (Millipore, 0.45 μm). After blocking in 5% nonfat milk in TBST (20 mM Tris-HCl pH 7.5, 137 mM NaCl, 0.1% (v/v) Tween 20) for 2 h at room temperature, the blots were, respectively incubated in appropriate primary antibodies at 4°C overnight. These were rabbit anti-TH antibody (ab112, Abcam, 1:200), rabbit anti-choline acetyltransferase antibody [EPR13024(B)] (ab181023, Abcam, 1: 1000), rabbit anti-β1-adrenergic receptor (ab3442, Abcam, 1:1000), mouse anti-M2 muscarinic receptor (ab2805, Abcam, 1:1000), and GAPDH (Cell Signaling Technology, 1:2500,). Then, membranes were washed with TBST × 3 and incubated with horseradish peroxidase-conjugated goat anti-rabbit secondary antibodies for 1 h at room temperature. After washing with TBST × 4, chemiluminescence signals were detected with SuperSignal Chemiluminescent Substrate (AB301B1/AB301A1, BIO vision), visualized with protein simple (Bio-Techne), and analyzed with the ImageJ system software.

### Immunofluorescence

The isolated fixed frozen hearts (n_sham_ = 5, n_pain_ = 5) were embedded in OCT medium, rapidly frozen, and cryosectioned into a series of 5-μm sections using a Leica 1850 cryostat (Leica CM1900, Germany). After heating at 60°C for 5 min, slices were permeabilized with 0.05% Triton-X100 in phosphate-buffered saline (PBS) for 10 min and washed for 3 × 10 min with PBS. Then, slides were blocked with 10% donkey serum for 1 h. Subsequently, for immunofluorescence, heart sections were incubated, respectively with primary antibodies against tyrosine hydroxylase (TH) (ab112, Abcam, 1:750), choline acetyltransferase (Chat) (ab181023, Abcam, 1:100), β1 (ab3442, Abcam, 1:100), and M2 (ab2805, Abcam, 1:200) overnight at room temperature. They were then washed for 3 × 10 min with PBS, and slides were incubated with appropriate secondary antibodies AlexaFluor 488 donkey anti-rabbit IgG (ab6721, Abcam, 1:1000) and AlexaFluor 568 goat anti-rat IgG (ab150153, Abcam, 1:1000) for 60 min at room temperature and washed for 3 × 10 min with PBS. Slides were sealed with a coverslip and observed with a fluorescence inverted microscope (Olympus CX41) and statistically analyzed using six slides for each group by the ImageJ software, ensuring at least one sample per heart was included in the analysis. Each section from a different left atrial sample was processed simultaneously, and the image conditions were the same to reduce variability among samples.

### Statistical analysis

The measurement data are all in the form of mean ± standard deviation. For comparing continuous measurement data, certificated normal distribution, the randomly non-paired *t*-test or the *F*-test was used, and for the counting data, the chi-squared test and Fisher's exact test were used. SPSS v22.0 software was used for statistical analysis. The results were plotted using GraphPad prism 6.0. *p-*Value of < 0.05 was considered statistically significant.

## Results

### Chronic pain induces the prolongation of PR intervals in mice

**Figure 1:**
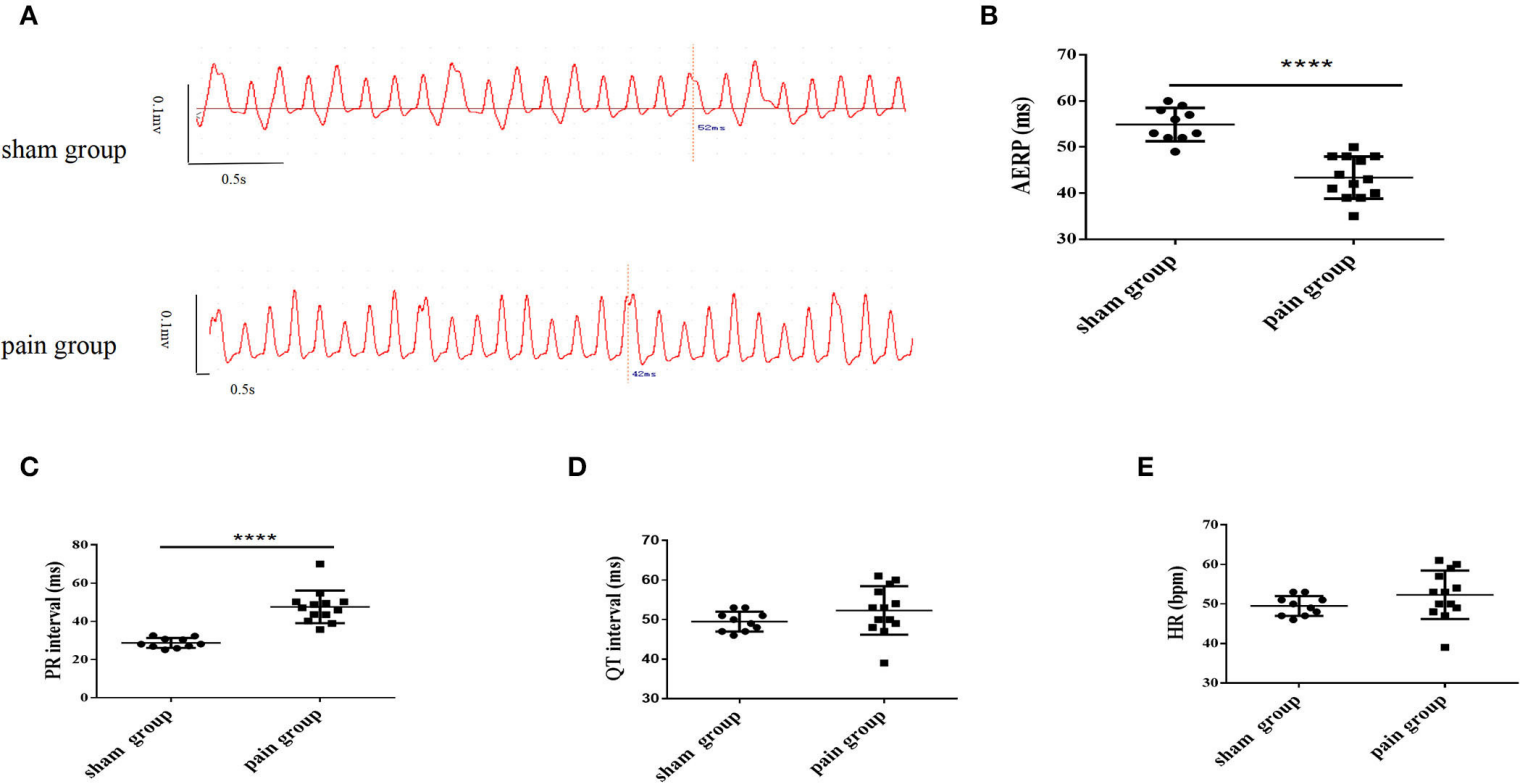
The measurement of left atrial bipolar cardiogram and basic characteristics of the electrocardiogram from lead II. **(A)** The left atrial effective refractory period (AERP) measured from the bipolar cardiogram (BCG) in sham and pain groups; **(B)** the AERP obtained from the BCG; the measurement of **(C)** PR interval, **(D)** QT interval, and **(E)** heart rate obtained from the surface ECG lead II in the sham and pain groups. The results are expressed as mean ± standard deviation. The scatter plot represents values from individual mice, with horizontal bars and error bars showing means and standard errors of the mean, respectively. **** represents *p* < 0.0001 (two-tailed Student's *t*-tests).

The chronic pain mice with successful CCI modeling (Supplementary Figure S1) that suffered constricting injury on the right sciatic nerve for 4 weeks displayed a longer PR interval (sham group: 28.69 ± 0.83 ms vs. pain group: 47.56 ± 2.37 ms, *p* < 0.0001) on ECG recordings under basal conditions, whereas other parameters (QT, HR) were not significantly different (Figures 1C–E and Table 1).

**Table 1:** The measurement of basic electrocardiograms in sham and pain groups.

	**Sham group**	**Pain group**	***p***
	**Mean ±SD (*n* = 10)**	**Mean ±SD (*n* = 13)**	
PR (ms)	28.69 ± 0.83	47.56 ± 2.37	<0.0001
QT (ms)	49.50 ± 0.79	52.31 ± 1.70	0.1888
HR (bpm)	458.8 ± 6.32	448.7 ± 13.22	0.5375

(Mean ± Standard Deviation).

### Chronic pain predisposes the mice to atrial fibrillation

**Figure 2:**
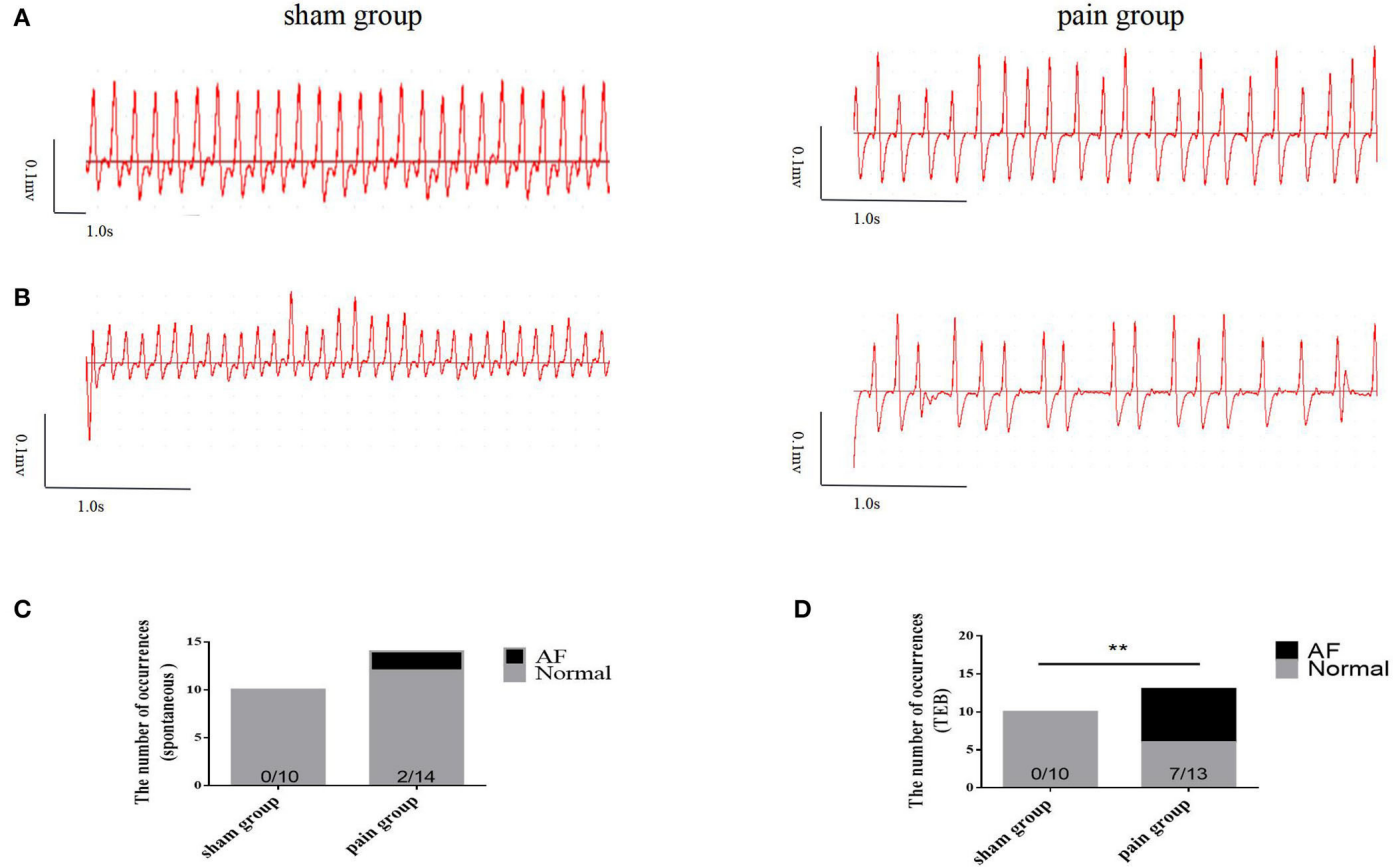
The susceptibility to atrial fibrillation. **(A)** The basic ECG in sham and pain groups; **(B)** the ECG after transesophageal burst pacing (TEB) in sham and pain groups; **(C)** the occurrence number of spontaneous AF before TEB in sham and pain groups; **(D)** the occurrence number of AF after TEB in sham and pain groups. The results are expressed as numbers and percentages. Histograms represent numbers and percentages. ** represents *p* < 0.01 (two-tailed Fisher's exact test).

Next, AF vulnerability was assessed. AF was defined as an episode of rapid and irregular atrial rhythm with a disordered ventricular response (irregular RR intervals) lasting at least 2 s. We found that approximately 15% of spontaneous AF episodes were observed (*n* = 2, 2/14) in CCI pain mice but not in sham control mice (*n* = 0, 0/10) before the transesophageal burst pacing (Figure 2C). After 5-min stabilization, one recovered sinus rate (paroxysmal AF, for 10 s), and the other showed persistent atrial fibrillation, which required atropine (0.5 mg/kg) to terminate. The latter mouse was removed from the study.

**Figure 3:**
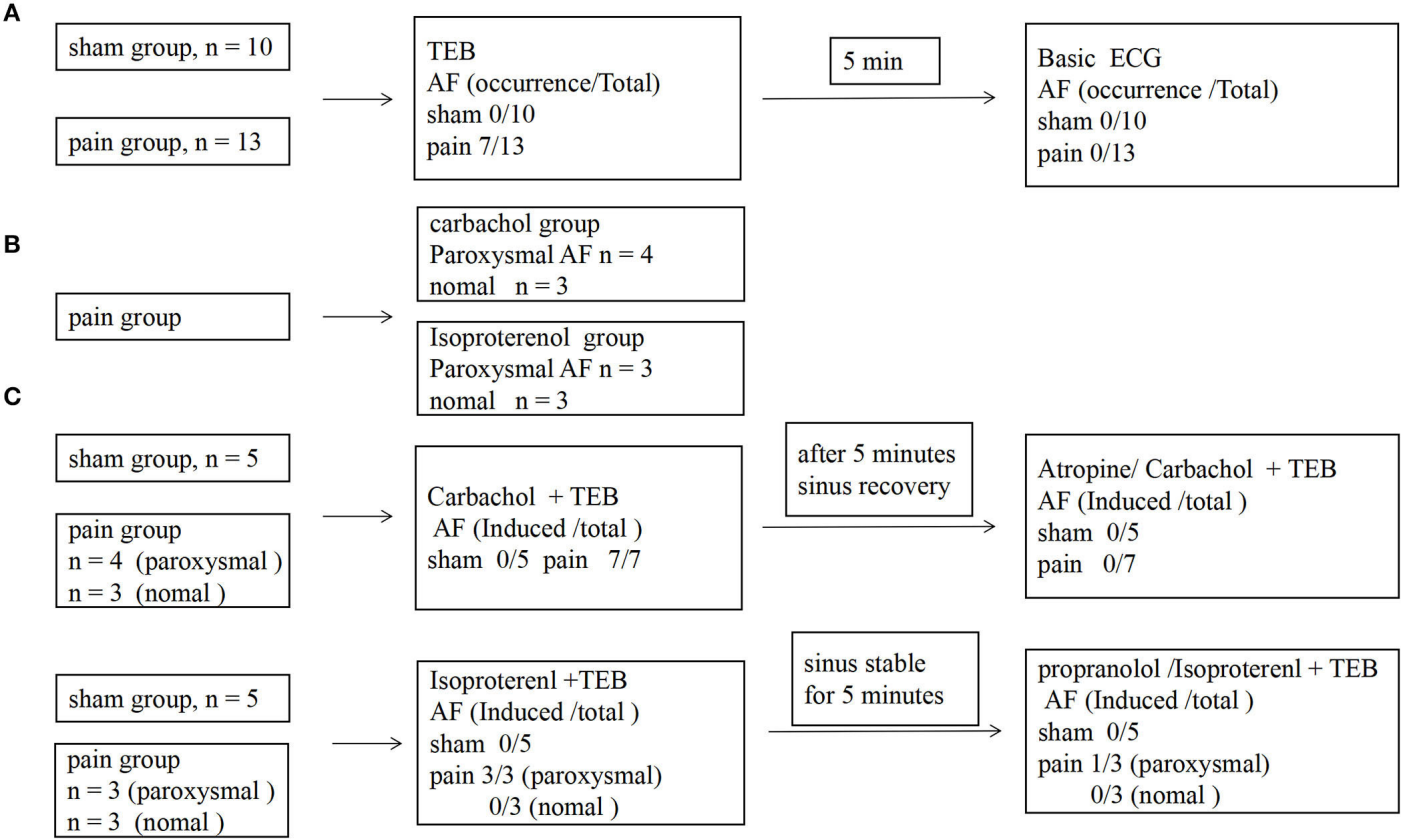
The protocol for investigating electrophysiological characteristics, atrial susceptibility, and the occurrence of atrial fibrillation. **(A)** The protocol and results of the AF susceptibility by transesophageal burst pacing (TEB) in sham and pain groups; **(B)** the grouping scheme of pain group; **(C)** the protocol and results of the effects of the autonomic nervous system in AF susceptibility between sham and pain groups.

Then, a transesophageal programmed stimulation at the esophageal position dorsal to the left atrial was performed to test the induction of AF. The result was that programmed burst pacing induced the occurrence of AF more in the pain group (*n* = 7, 7/13) than in the sham group (*n* = 0, 0/10) (Figures 2A,B,D, 3A). The increased AF propensity by CCI was further substantiated *in vitro* in Langendorff perfused hearts, in which programmed S1S2 stimulation induced a shortening of AERP as compared to that in sham hearts (sham group: 54.90 ± 1.14 ms vs. pain group: 43.38 ± 1.26 ms, *p* < 0.0001) (Figures 1A,B and Table 2). Together, these data indicated that chronic pain increases the vulnerability of AF in mice.

**Table 2:** The measurement of electrophysiological characteristics in the Langendorff perfusion model in sham and pain groups.

	**Shamgroup**	**Pain group**	***p***
	**Mean ±SD (*n* = 10)**	**Mean ±SD (*n* = 13)**	
AERP (ms)	54.90 ± 1.14	43.38 ± 1.26	<0.0001

(Mean ± Standard Deviation).

### Muscarinic receptor overactivation mediates the chronic pain-induced AF

**Figure 4:**
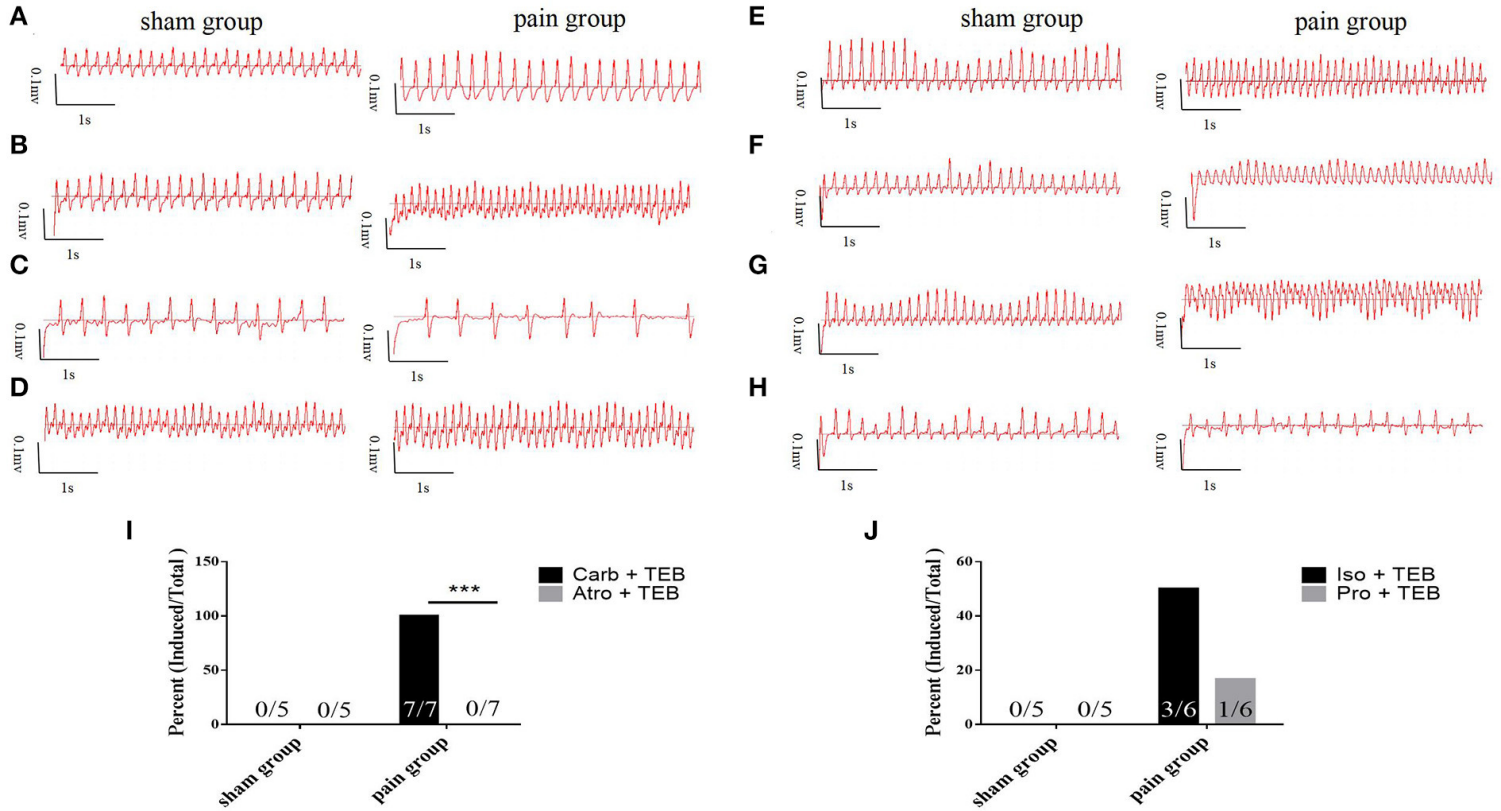
Vagal effects **(A**–**D)** and sympathetic effects **(E**–**H)** on atrial fibrillation. **(A)** The basic ECG in sham and pain groups; **(B)** the ECG after transesophageal burst pacing (TEB) in sham and pain groups; **(C)** the ECG after TEB with carbachol pretreatment in sham and pain groups; **(D)** the ECG after atropine/carbachol and TEB in sham and pain groups; **(E)** the basic ECG in sham and pain groups; **(F)** the ECG after TEB in sham and pain groups; **(G)** the ECG after TEB with isoproterenol pretreatment in sham and pain groups; **(H)** the ECG after propranolol/isoproterenol and TEB in sham and pain groups; **(I)** the AF induced percent pretreated, respectively with carbachol and atropine/carbachol after TEB in sham and pain groups; and **(J)** the AF induced percent pretreated, respectively with isoproterenol and propranolol/isoproterenol after TEB in sham and pain groups. The results are expressed as numbers and percentages. Histograms represent percent. *** represents *p* < 0.001 (two-tailed Fisher's exact test).

We went on to ask whether chronic pain-induced AF involves the changed activity of sympathetic or parasympathetic nerves. Carbachol, a nonselective muscarinic receptor agonist increases AF occurrence (3, 16, 17, 19), significantly increased AF susceptibility with transesophageal burst pacing in the pain group (*n* = 7, 7/7), consistent with enhanced cholinergic sensitivity in CCI mice. Administration of atropine, a muscarinic receptor blocker, almost completely prevented the induction of AF by transesophageal burst pacing in the pain group (*n* = 0, 0/7) (7/7 vs. 0/7, *p* = 0.0006 <0.001) (Figures 3B,C, 4). These results indicated that cholinergic receptor activation was important in enhancing AF vulnerability in CCI mice.

The potential contribution of sympathetic activity in AF inducibility was also examined. In CCI and sham control mice subjected to transesophageal burst pacing, intraperitoneal injection of isoproterenol, a noradrenergic β1 and β2 receptor agonist, did not affect the AF susceptibility in the sham (*n* = 0, 0/5) or the pain groups (*n* = 3, 3/6) which showed paroxysmal AF induced by TEB alone. After 5-min stabilization, recovery sinus rate, propranolol, followed by isoproterenol, did not significantly enhance AF susceptibility with TEB in sham (*n* = 0, 0/5) or the pain groups (*n* = 1, 1/6) (Figures 3B,C, 4).

At the same time, the noradrenergic α1 receptor arrhythmogenic effect by pretreatment α-blocker phentolamine and found that it has no effect on the AF occurrence by transesophageal burst pacing in sham (*n* = 0, 0/5) or the pain groups (*n* = 5, 5/5) (Supplementary Figure S2).

Collectively, the AF prompting effects of chronic pain in mice were dependent on parasympathetic overactivation, as atropine completely eliminates the AF inducibility.

Chronic pain induces the increase of muscarinic acetylcholine receptor-2 expression.

**Figure 5:**
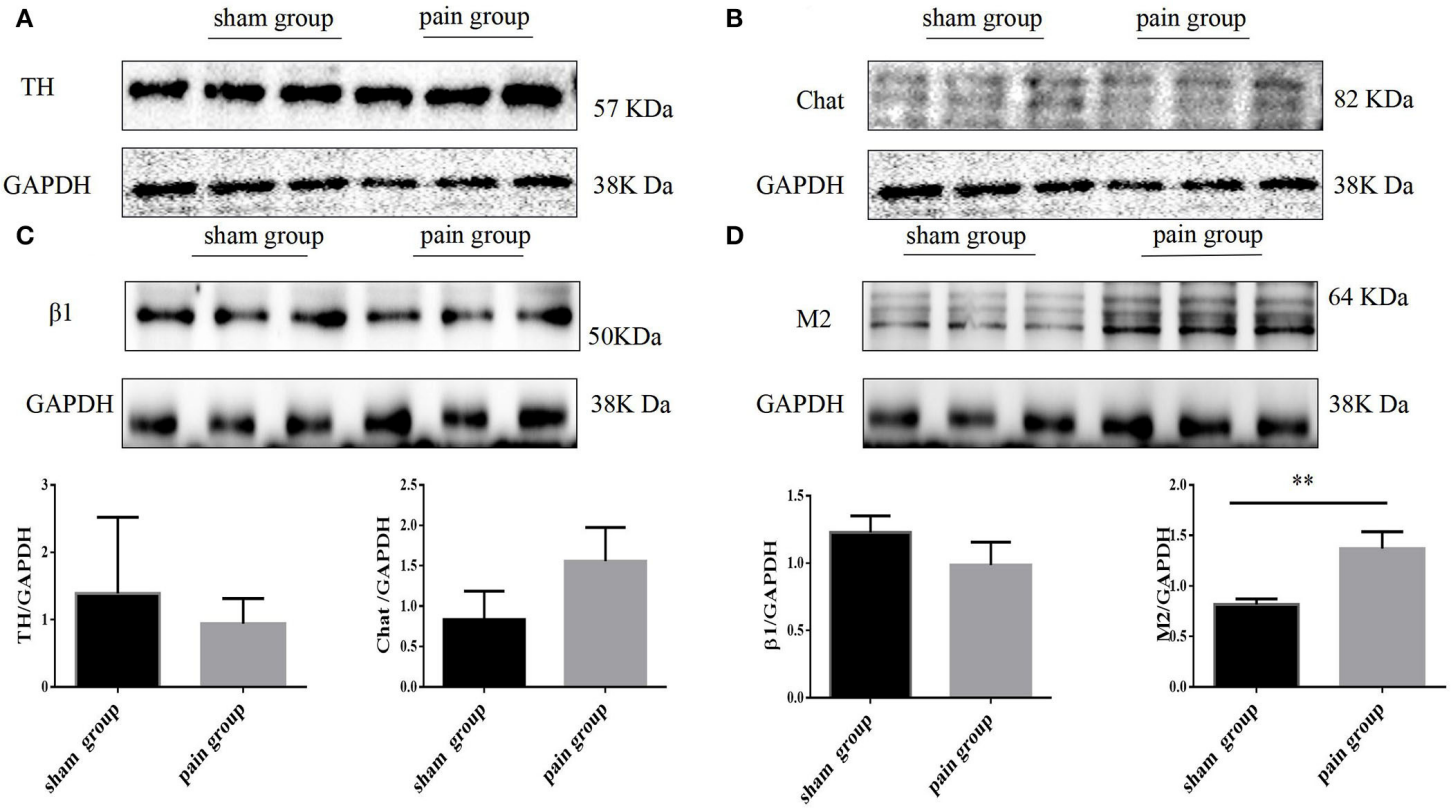
Autonomic nervous system related protein expression by Western blot. **(A)** Tyrosine hydroxylase (TH), **(B)** choline acetyltransferase (Chat), **(C)** adrenergic β1 receptor, and **(D)** muscarinic acetylcholine M2 receptor expression with Western blot in sham and pain groups. The results are expressed as mean ± standard deviation. The histograms represent values. ** represents *p* < 0.01 (two-tailed Student's *t*-tests).

To evaluate the expression of neural remodeling proteins, we used Western blot and immunofluorescence to analyze the expression of autonomic nervous receptors. Anti-tyrosine hydroxylase (TH) and anti-choline acetyltransferase (Chat) were further applied to investigate the distribution and quantity of adrenergic and cholinergic neurons. The results showed that the M2 AChR expression increased more significantly in the pain group (1.89 ± 0.34 vs. 0.13 ± 0.02, *p* = 0.0067 <0.05), and there were no differences in the distribution of adrenergic and cholinergic neurons, or in β1 receptor expression (Figure 5). The M2 AChR membrane protein expression was applied to investigate the distribution of M2 AChR distribution on the cell membrane, which found more expression in the pain group (1.47 ± 0.09 vs. 0.95 ± 0.14, *p* = 0.0378 <0.05) (Supplementary Figure S3).

**Figure 6:**
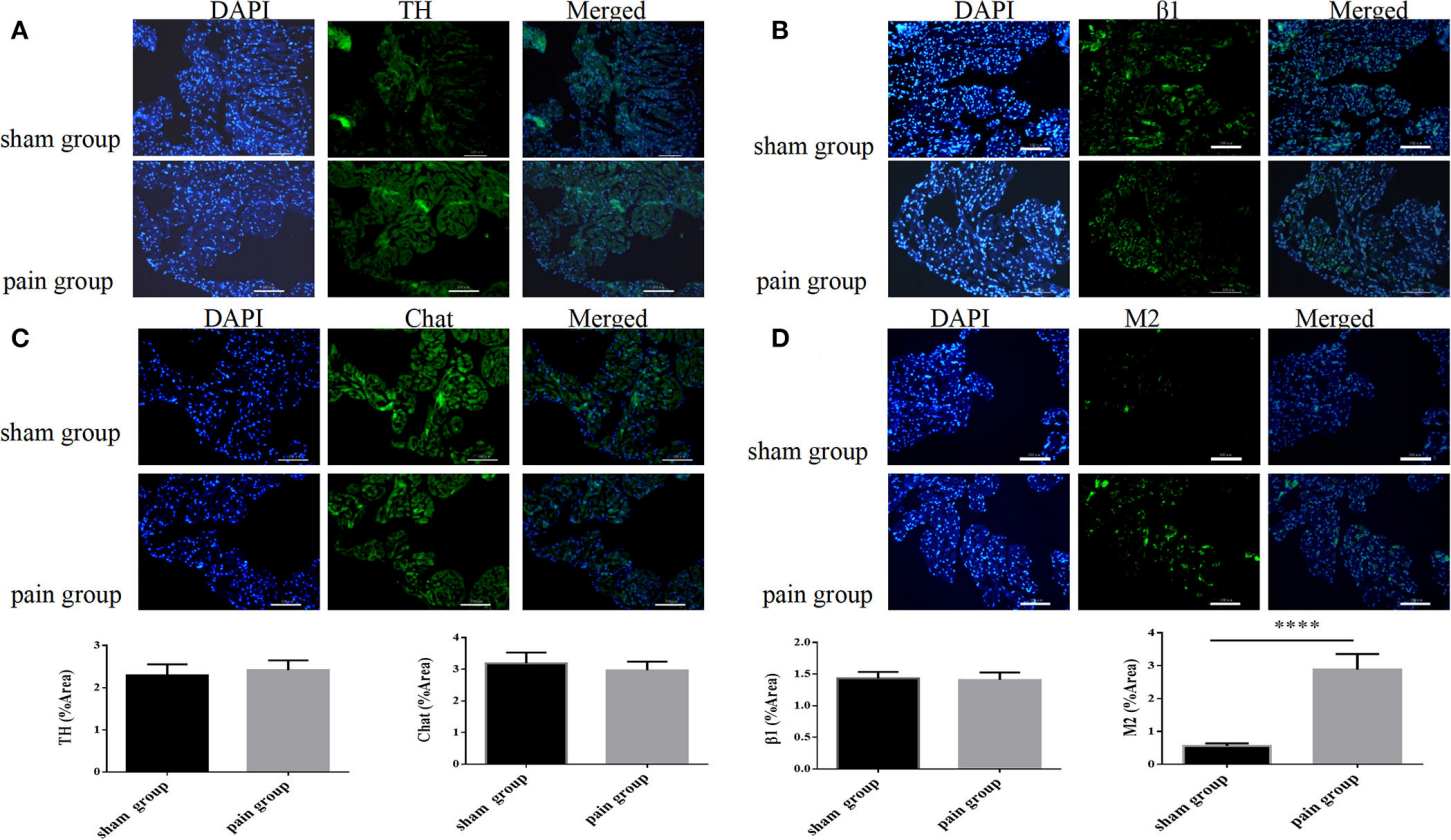
Autonomic nervous system-related protein expression distribution by immunofluorescence. **(A)** Tyrosine hydroxylase (TH), **(B)** choline acetyltransferase (Chat), **(C)** adrenergic β1 receptor, and **(D)** muscarinic acetylcholine M2 receptor expression distribution with immunofluorescence in sham and pain groups. The results are expressed as mean ± standard deviation. The histograms represent values. **** represents *p* < 0.0001 (two-tailed Student's *t*-tests).

The immunofluorescence results were consistent with the Western blot findings. We found that the M2 expression increased more significantly in the pain group (0.56 ± 0.03 vs. 2.89 ± 0.18, *p* < 0.0001), and there were no significant differences in TH, Chat, or β1 expressions (Figure 6).

RGS4, the M2 receptor signaling cascade, was investigated by Western blot, which showed that the RGS4 expression was more in the pain group (0.87 ± 0.05 vs. 0.51 ± 0.03, *p* = 0.0035 <0.005) (Supplementary Figure S4).

## Discussion

We found that mice with chronic pain were substantially more susceptible to AF provoked by TEB pacing than those without chronic pain. This was confirmed by electrophysiological characteristics: the PR interval was longer indicating slower atrial conduction; the AERP was shorter indicating that atrial substrates in these animals may be more prone to arrhythmias.

Numerous possible factors may contribute to the relationship between chronic pain and cardiovascular disorders (31–33). Cardiovascular risk factors, metabolic syndromes (such as high body mass index, and high serum triglycerides), inflammation (such as increase in C-reactive protein and fibrinogen), and lifestyle (such as smoking, sedentary behavior, and increasing age) are associated with chronic pain (31–33). Werner et al. (14) noted that patients with chronic non-cancer pain had many complex confounding risk factors, such as inactivity, respiratory diseases, and metabolic disorders. Our study excluded those interfering factors by establishing a valid animal model.

It is known that parasympathetic activation is a factor in AF (16, 34). Yu Jin et al. (22) reported that cardiovascular function was mainly regulated through parasympathetic tone 2 weeks after CCI. Chronic intermittent hypoxia worsened AF susceptibility in rat with enhanced parasympathetic sensitivity, reflected in increased cholinergic responses, and increased M2 muscarinic receptors (35).

For the first time, our study found that the cholinergic nervous system had a noticeable role in enhancing AF sensitivity in mice with chronic pain. The occurrence of AF was driven by enhanced atrial cholinergic responses, accompanied by greater M2 AChR expression. M2 AChR is a plasma membrane protein and performs physiological functions on the cell membrane. The M2 AChR on the plasma membrane, indicating the distribution on the cell membrane, was found more expression in the pain group. Many studies have shown that cholinergic signaling increased atrial I_k−Ach_ activity, thus enhancing spatial dispersion of refractoriness, and this might be a potential mechanism in chronic pain (17, 19, 36). Our study found that the shortening of AERP might be consistent with this hypothesis, although a single-point measurement. Sympathetic nervous system activity also can promote AF, and catecholamine can facilitate cholinergically induced AF (37). The noradrenergic might contribute to AF vulnerability in CCI chronic pain mice, as shown in our research β-receptor blocker propranolol preventing TEB induction AF occurrence in one mouse, although these data failed to reach statistical significance. α1 receptor is a G-protein-coupled receptor and causes Ca^2+^ release from SR by generating IP3 and DAD, which thought may contribute to atrial fibrillation (20, 38, 39). There was no effect on the AF occurrence between the two groups by pretreatment of α-blocker phentolamine. These results might suggest that parasympathetic activation is more important than sympathetic activation for inducing AF susceptibility in CCI chronic pain mice. Regulators of G-protein signaling 4 (RGS4) is the M2 receptor signaling cascade to deactivate G-protein signaling and damp down the parasympathetic input. It was known that RGS4 was expressed more in the SA node than in the atrial. RGS4 absence results in a predisposition to atrial fibrillation through the IP3-Ca^2+^ release way (40, 41). Our research showed that the RGS4 of atrial cell expression was more in CCI chronic pain mice, which might be one of the compensatory mechanisms in the development of chronic pain-related AF that deserves our future study.

It was a fact that there was no significant difference between groups in basic heart rate and some pain mice even have faster heart rates than sham ones, which might suggest that M2R various may have less effect on the sinoatrial note. We have investigated the ECG trace of the first few AF beats afterward burst pacing, which performs less morphological variation in P-wave. It might indicate that the mechanism of chronic pain-related AF was the focal source. Additionally, we should investigate the atrial structure remodeling in our further research. Limitations of our study were small sample size, no female animals, and the electrophysiological parameter AERP should have been measured *in vivo* and in multiple parts of the atrial. We cannot completely preclude the potential influence of pentobarbital on the autonomic nerve, despite the impact may be weak. The M2 AChR atria spatial expression dispersion is a significantly possible mechanism of chronic pain-related AF. So, we should investigate this in our next research. The M2 AChR expression in chronic pain mice atrial is an important observation in our research, which should be confirmed in the M2RKO mouse model in further experiments. We established a chronic pain CCI animal model for simulating the emotion-related chronic stress-induced associated with AF. It will be interesting to evaluate the chronic pain mice emotional situation in our model and further investigate the neural remodeling in chronic stress-induced pain and AF. Many studies have reported that cardiomyocytes are able to synthesize and release acetylcholine and compose the non-neuronal cholinergic system (NNCS), which can regulate energy metabolism, sarcoplasmic reticulum Ca^2+^ mobilization, and local inflammatory responses with the neuronal cholinergic system (42–44). NNCS may play an important role in chronic pain-related AF. We should do further research on this issue.

Our study has shown that wild-type C57BL/6J male mice with chronic pain from CCI were more prone to AF and had increased susceptibility to AF after treatment with carbachol. We found that the atria expressing more M2 receptors after chronic pain develops may be a novel mechanism of AF susceptibility.

## Data availability statement

The original contributions presented in the study are included in the article/Supplementary material, further inquiries can be directed to the corresponding author/s.

## Ethics statement

This animal study was reviewed and approved by Nanjing Medical University Animal Ethics Committee (ethical code: IACUC-1808001).

## Author contributions

CG contributed to the conception and design of the work. YD, FL, SW, XT, HD, WH, XY, and LZ contributed to the acquisition, analysis, or interpretation of data for the work. SL and JL contributed to the drafting of the work or revising it critically for important intellectual content. All authors approved the final version of the manuscript and agree to be accountable for all aspects of the work. All persons designated as authors qualify for authorship and all those who qualify for authorship are listed. All authors contributed to the article and approved the submitted version.

## Funding

This study was supported by the National Natural Science Foundation of China (Grant Number 81570292).

## Conflict of interest

The authors declare that the research was conducted in the absence of any commercial or financial relationships that could be construed as a potential conflict of interest.

## Publisher's note

All claims expressed in this article are solely those of the authors and do not necessarily represent those of their affiliated organizations, or those of the publisher, the editors and the reviewers. Any product that may be evaluated in this article, or claim that may be made by its manufacturer, is not guaranteed or endorsed by the publisher.

## Supplementary material

Supplementary Figure S1 Nociceptive behaviors in sham and pain groups were evaluated by the von Frey filament test applied on the right paw before chronic constriction injury to the sciatic nerve, and 4 weeks later. The percentage of paw withdrawal frequency in sham and pain groups. ^****^ represents *p* < 0.0001 (two-way ANOVA).

Supplementary Figure S2 The adrenergic α1 receptor effect on atrial fibrillation. **(A)** The basic ECG in sham and pain groups; **(B)** the ECG after transesophageal burst pacing (TEB) in sham and pain groups; **(C)** the ECG after TEB with phentolamine pretreatment in sham and pain groups. The results are expressed as numbers and percentages. Histograms represent percent (two-tailed Fisher's exact test).

Supplementary Figure S3 The adrenergic β1 receptor and M2 ACh receptor expression on the cell plasma membrane by Western blot. **(A)** Adrenergic β1 receptor and **(B)** muscarinic acetylcholine M2 receptor expression on the plasma membrane with Western blot in sham and pain groups. The results are expressed as mean ± standard deviation. The histograms represent values. ^*^represents *p* < 0.05 (two-tailed Student's *t*-tests).

Supplementary Figure S4 The RGS4 protein expression by Western blot in sham and pain groups. The results are expressed as mean ± standard deviation. The histograms represent values. ^**^represents *p* < 0.005 (two-tailed Student's *t*-tests).
