# Supplementary material for: Muscarinic receptor regulation of chronic pain-induced atrial fibrillation
Source: Front Cardiovasc Med. 2022 Sep 15;9:934906. doi: 10.3389/fcvm.2022.934906 (PMC9521049; doi:10.3389/fcvm.2022.934906)

TH TH GAPDH


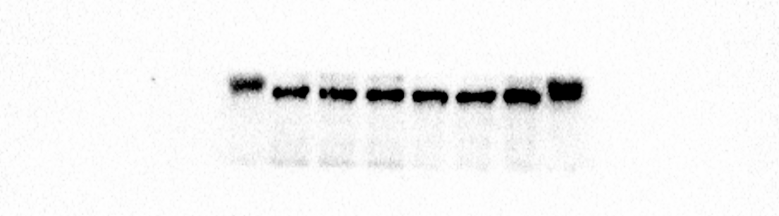


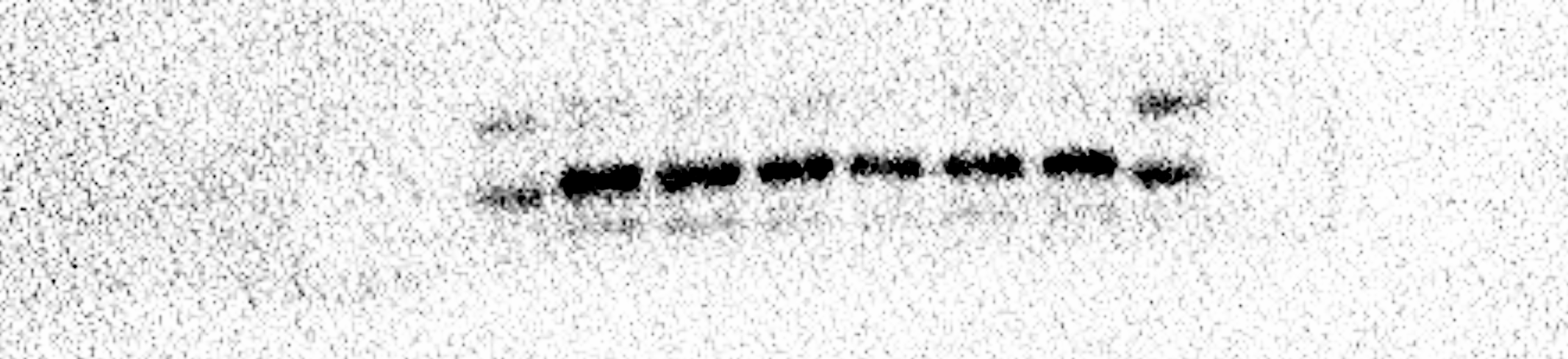


Chat Chat GAPDH


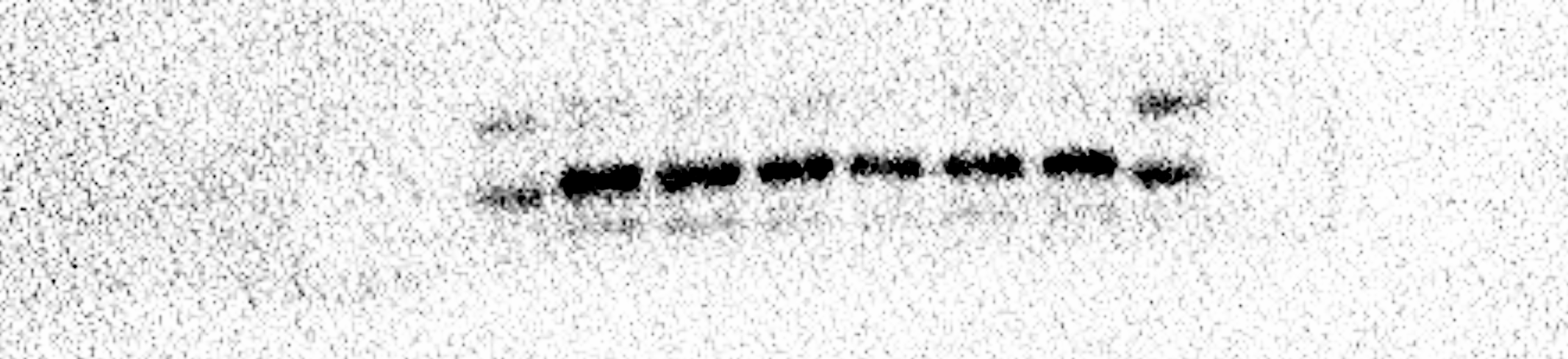


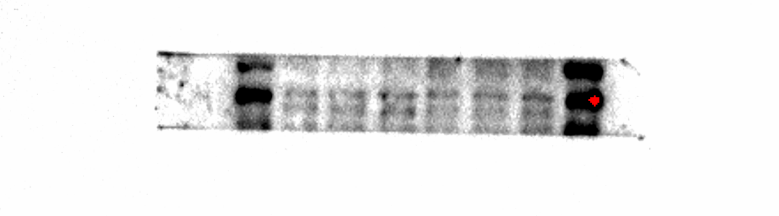


β1 (bs-20177R, Bio, 1:2000) β1 GAPDH


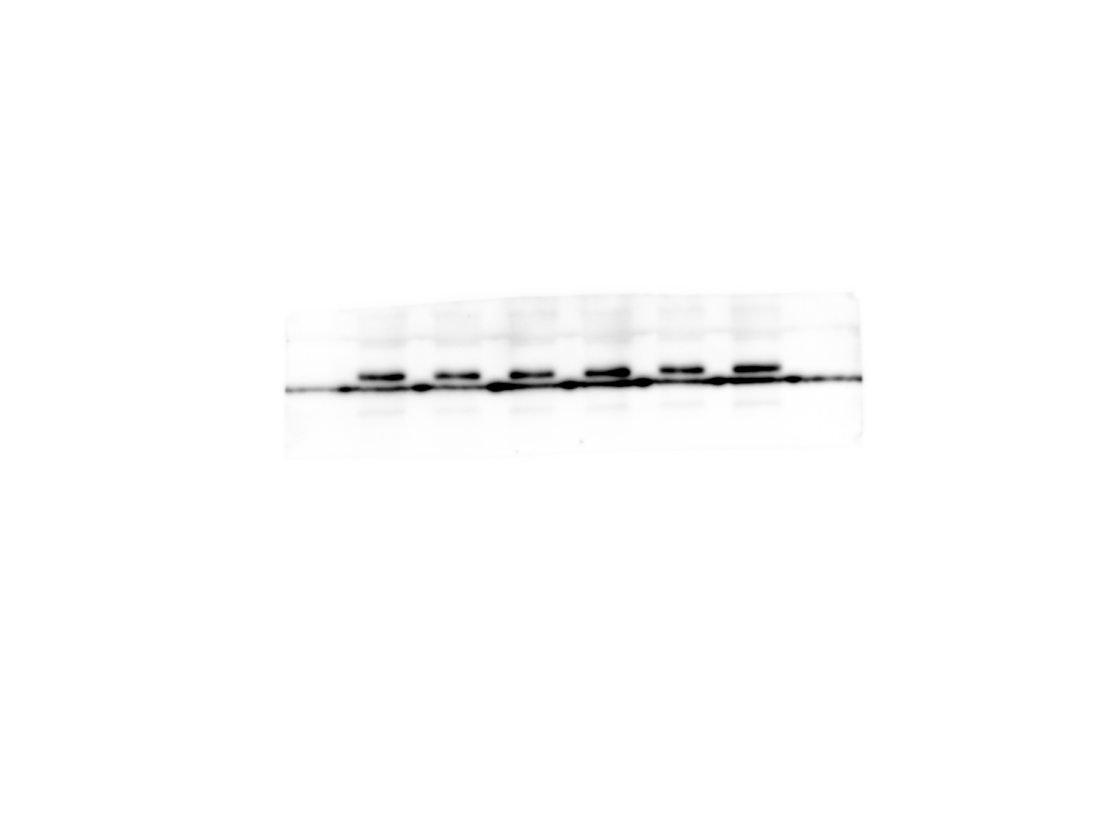


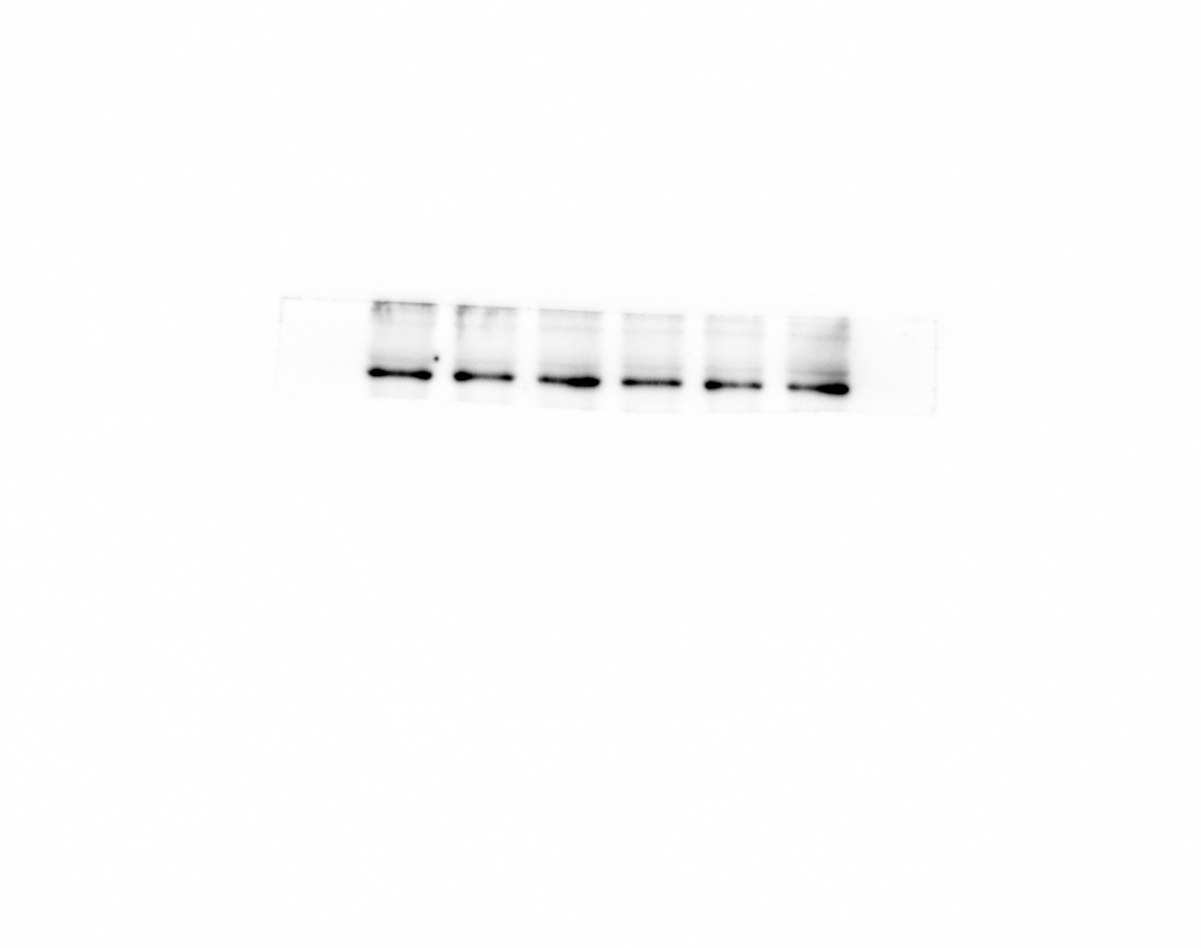


β1 (ab3442, Abcam, 1:1000) β1GAPDH


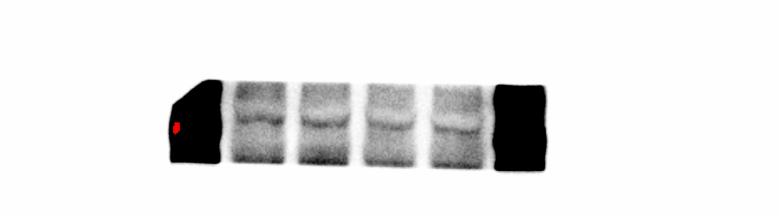


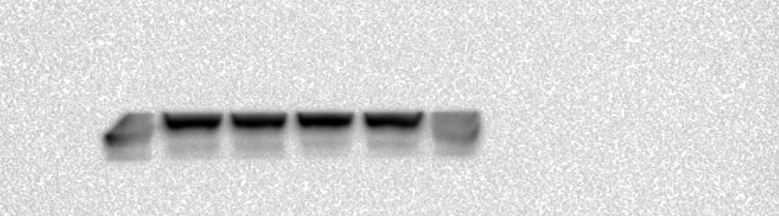


M2 ( bs-0441R, Bio, 1:2000) M2 GAPDH


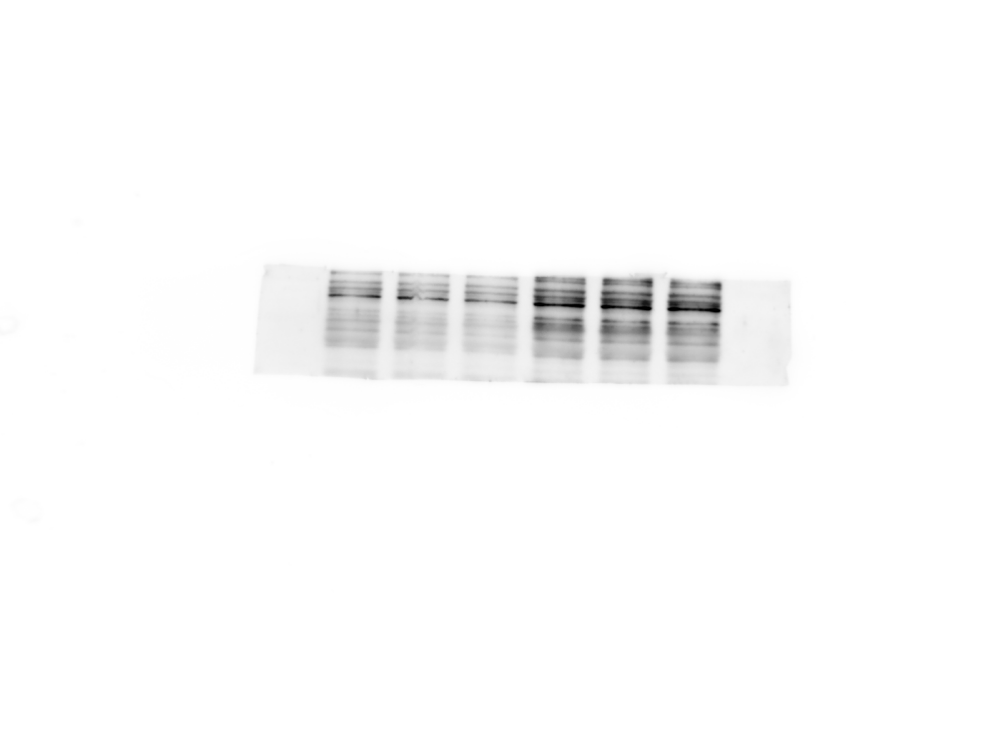


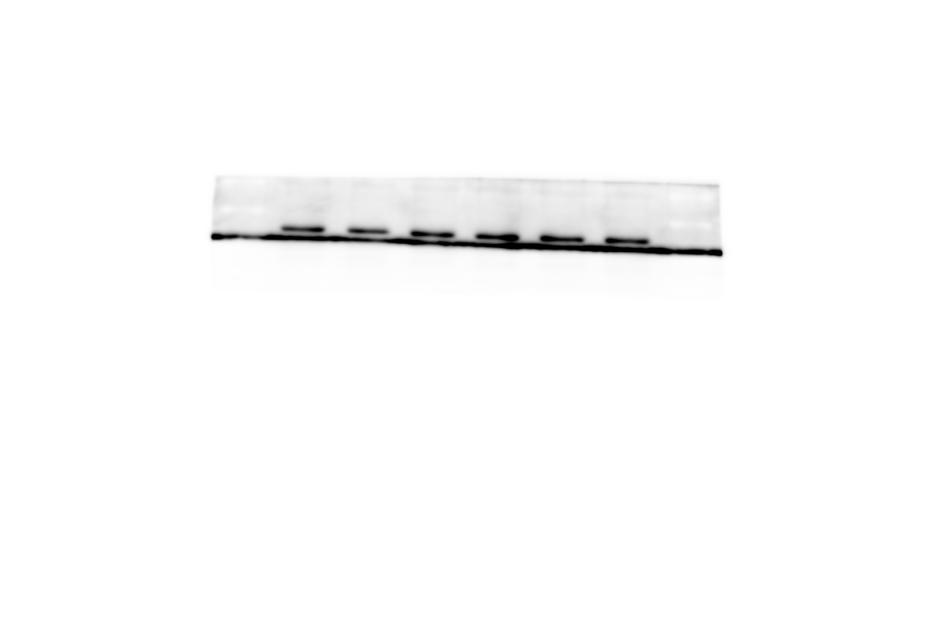


M2 (ab2805, Abcam, 1:1000) M2 GAPDH


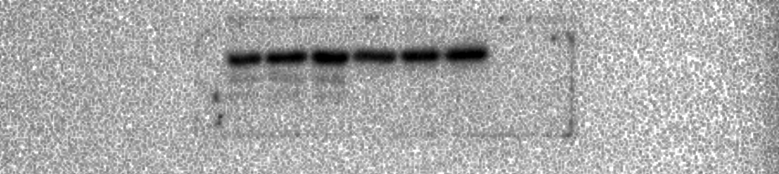

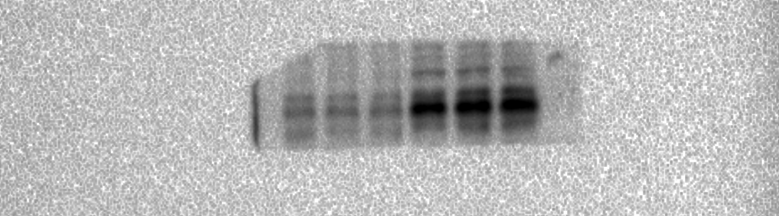


RGS4 RGS4 GAPDH


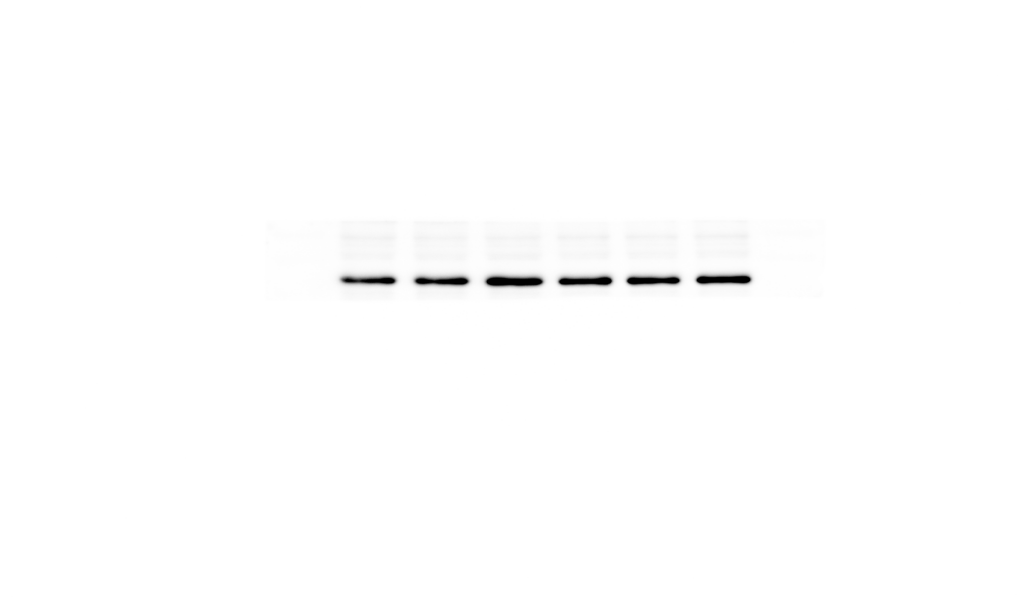


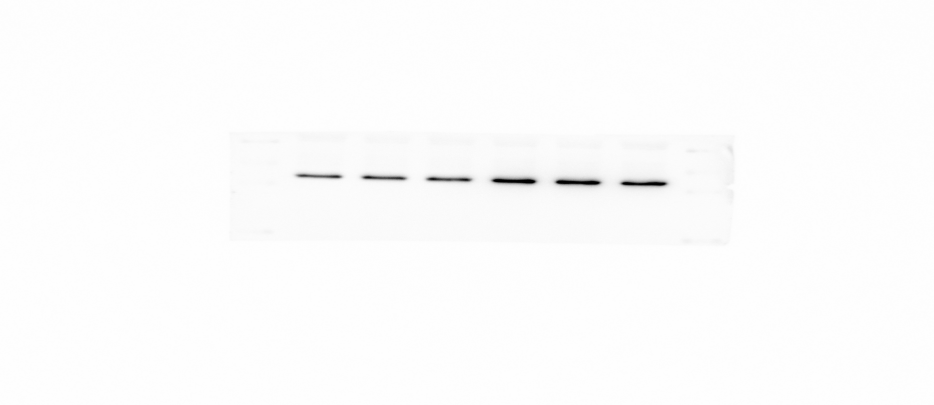


β1 β1 Na-K-ATP


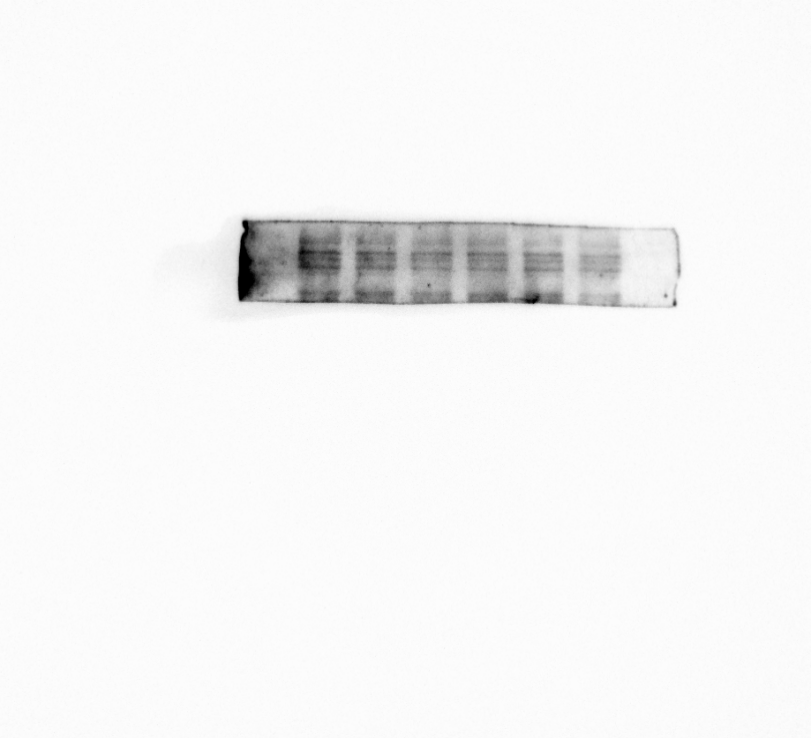

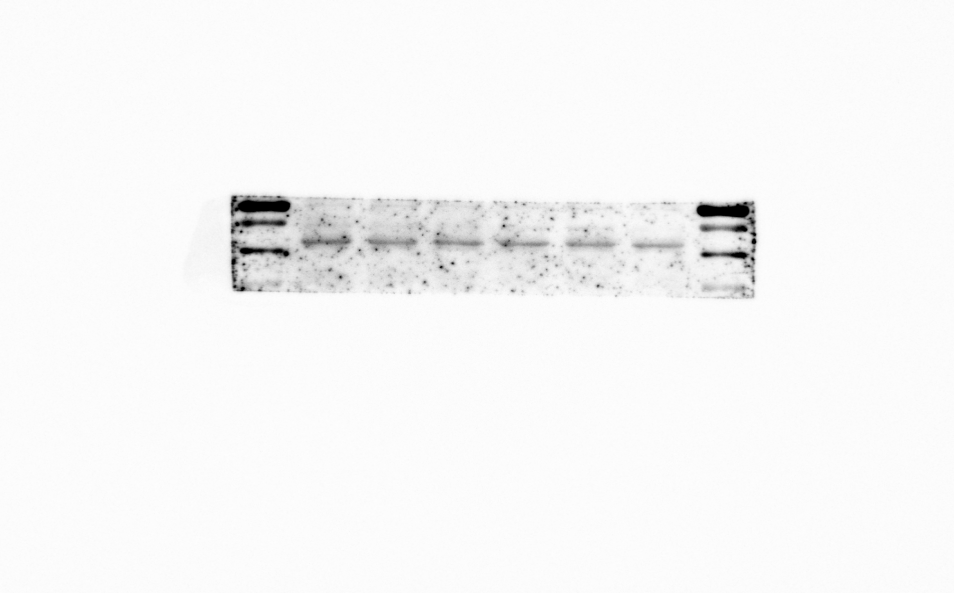


M2 M2 Na-K-ATP


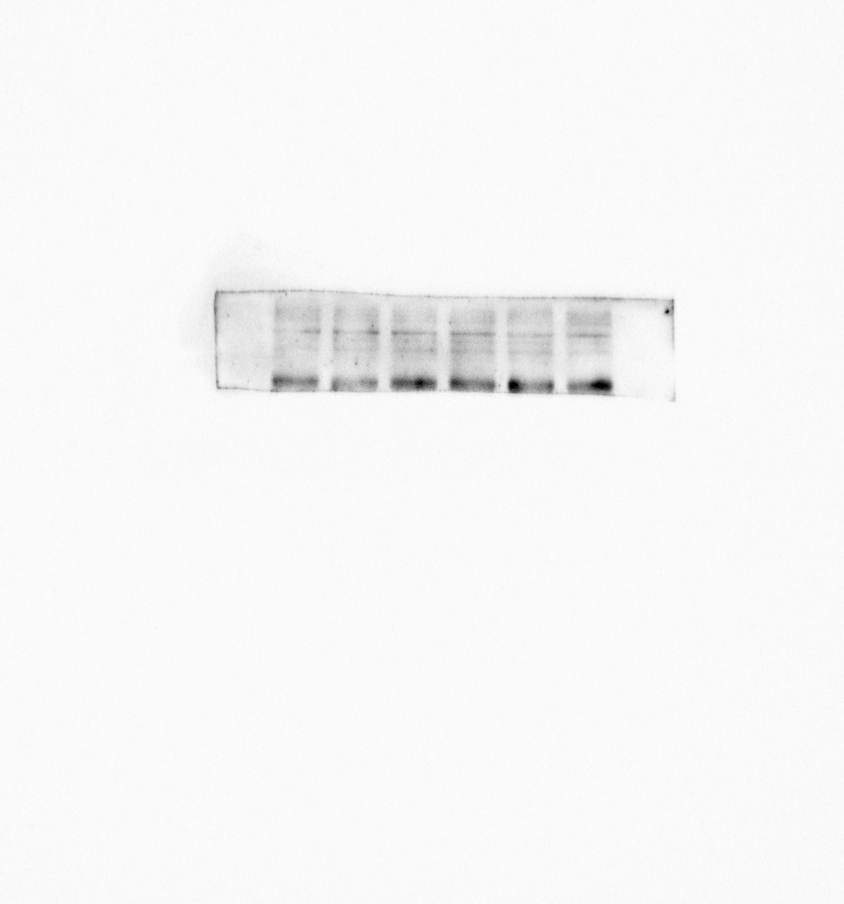

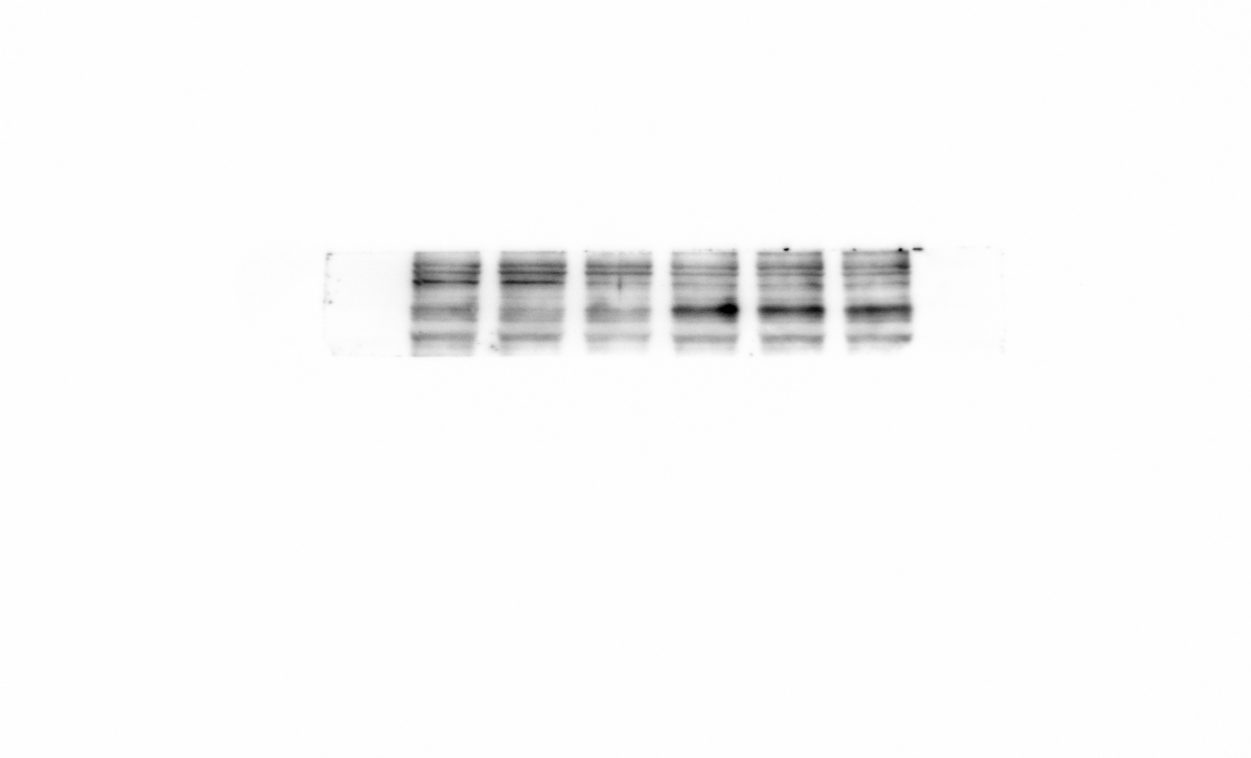

Supplement: Supplementary file 2 [file Data_Sheet_2.docx]
