# Supplementary figures and images for: Muscarinic receptor regulation of chronic pain-induced atrial fibrillation
Source: Front Cardiovasc Med. 2022 Sep 15;9:934906. doi: 10.3389/fcvm.2022.934906 (PMC9521049; doi:10.3389/fcvm.2022.934906)

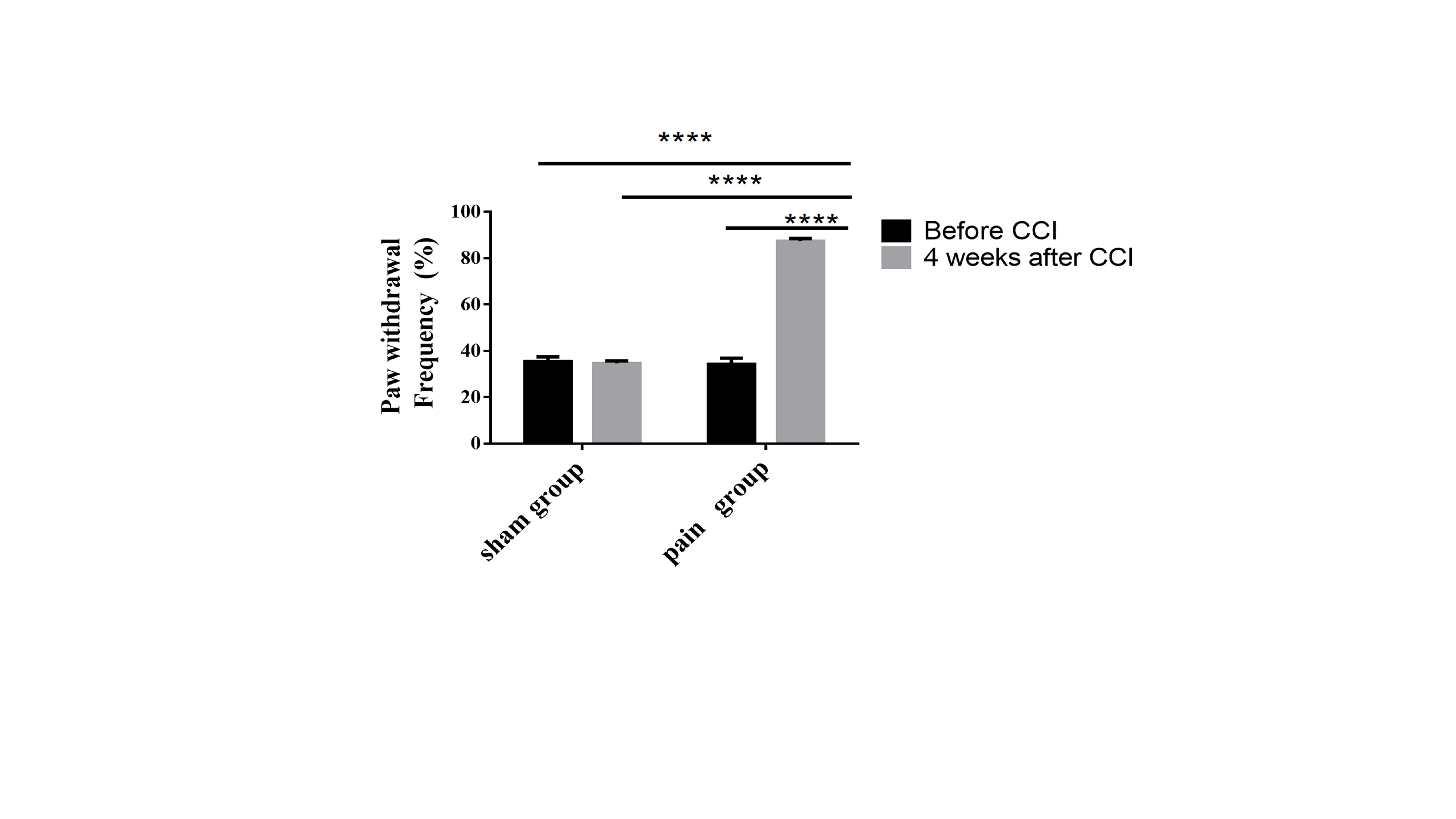

Supplement: Supplementary Figure S1 — Nociceptive behaviors in sham and pain groups were evaluated by the von Frey filament test applied on the right paw before chronic constriction injury to the sciatic nerve, and 4 weeks later. The percentage of paw withdrawal frequency in sham and pain groups. **** represents p < 0.0001 (two-way ANOVA). [file Image_1.JPEG]

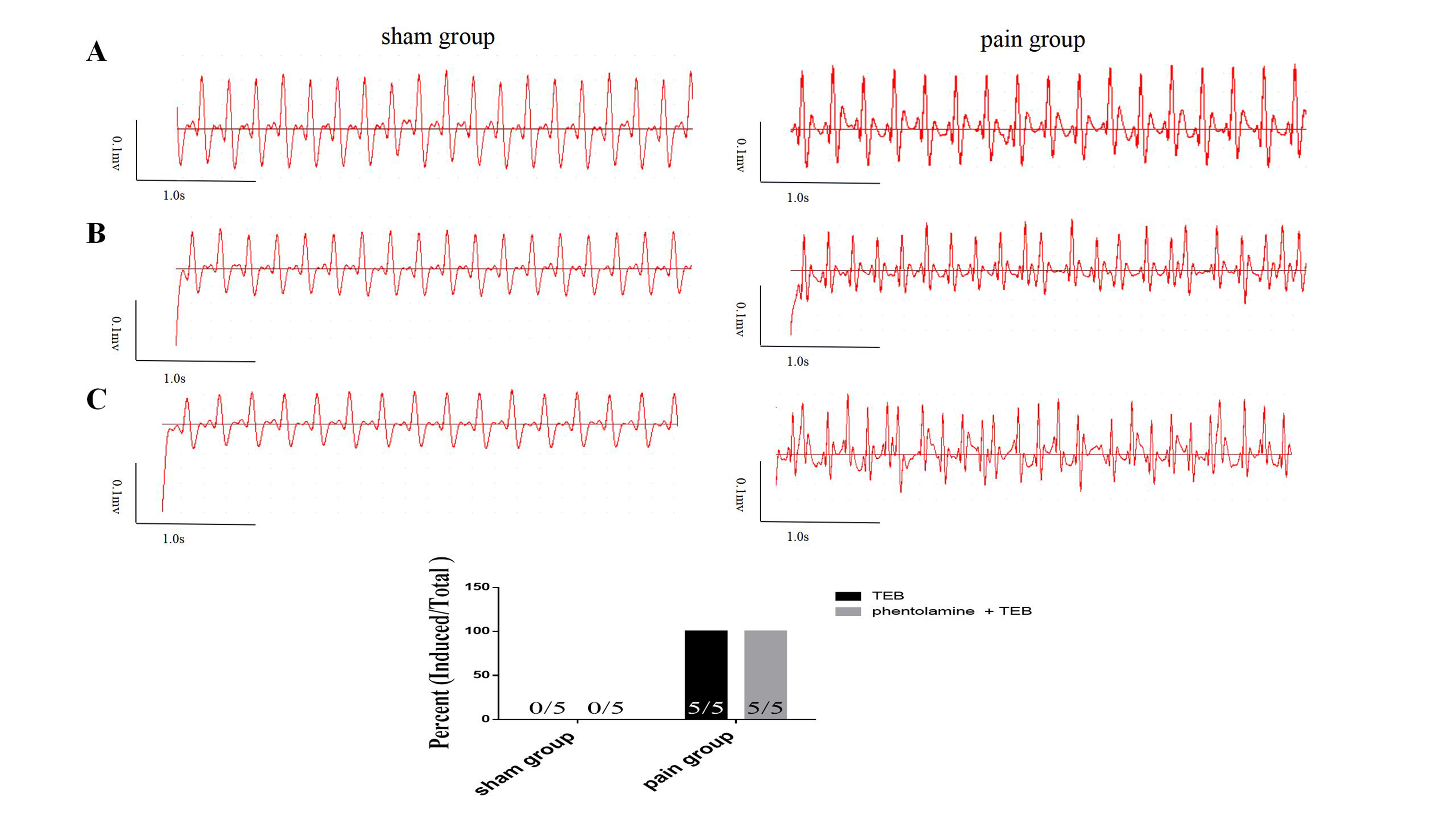

Supplement: Supplementary Figure S2 — The adrenergic α1 receptor effect on atrial fibrillation. (A) The basic ECG in sham and pain groups; (B) the ECG after transesophageal burst pacing (TEB) in sham and pain groups; (C) the ECG after TEB with phentolamine pretreatment in sham and pain groups. The results are expressed as numbers and percentages. Histograms represent percent (two-tailed Fisher's exact test). [file Image_2.JPEG]

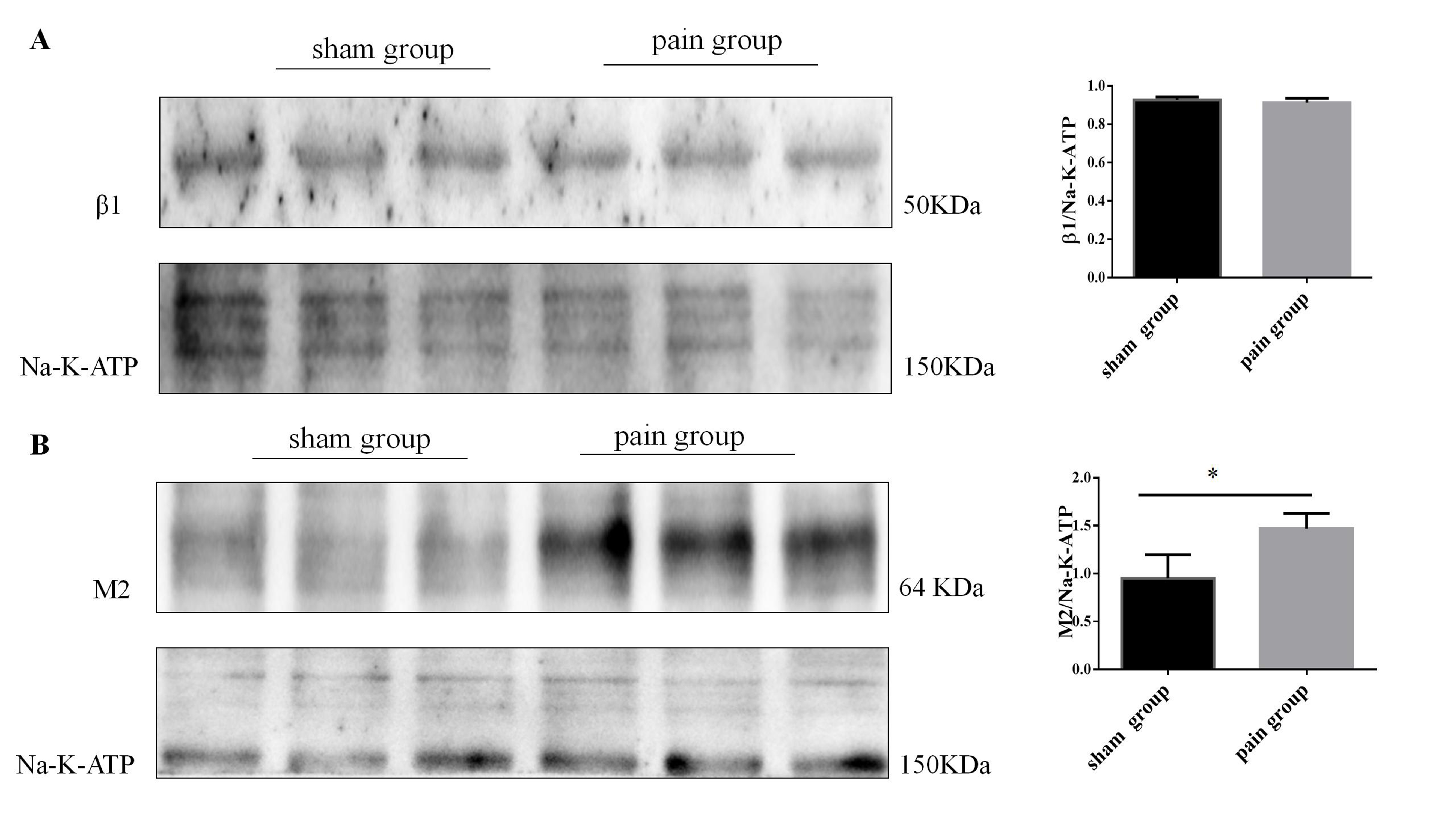

Supplement: Supplementary Figure S3 — The adrenergic β1 receptor and M2 ACh receptor expression on the cell plasma membrane by Western blot. (A) Adrenergic β1 receptor and (B) muscarinic acetylcholine M2 receptor expression on the plasma membrane with Western blot in sham and pain groups. The results are expressed as mean ± standard deviation. The histograms represent values. *represents p < 0.05 (two-tailed Student's t-tests). [file Image_3.JPEG]

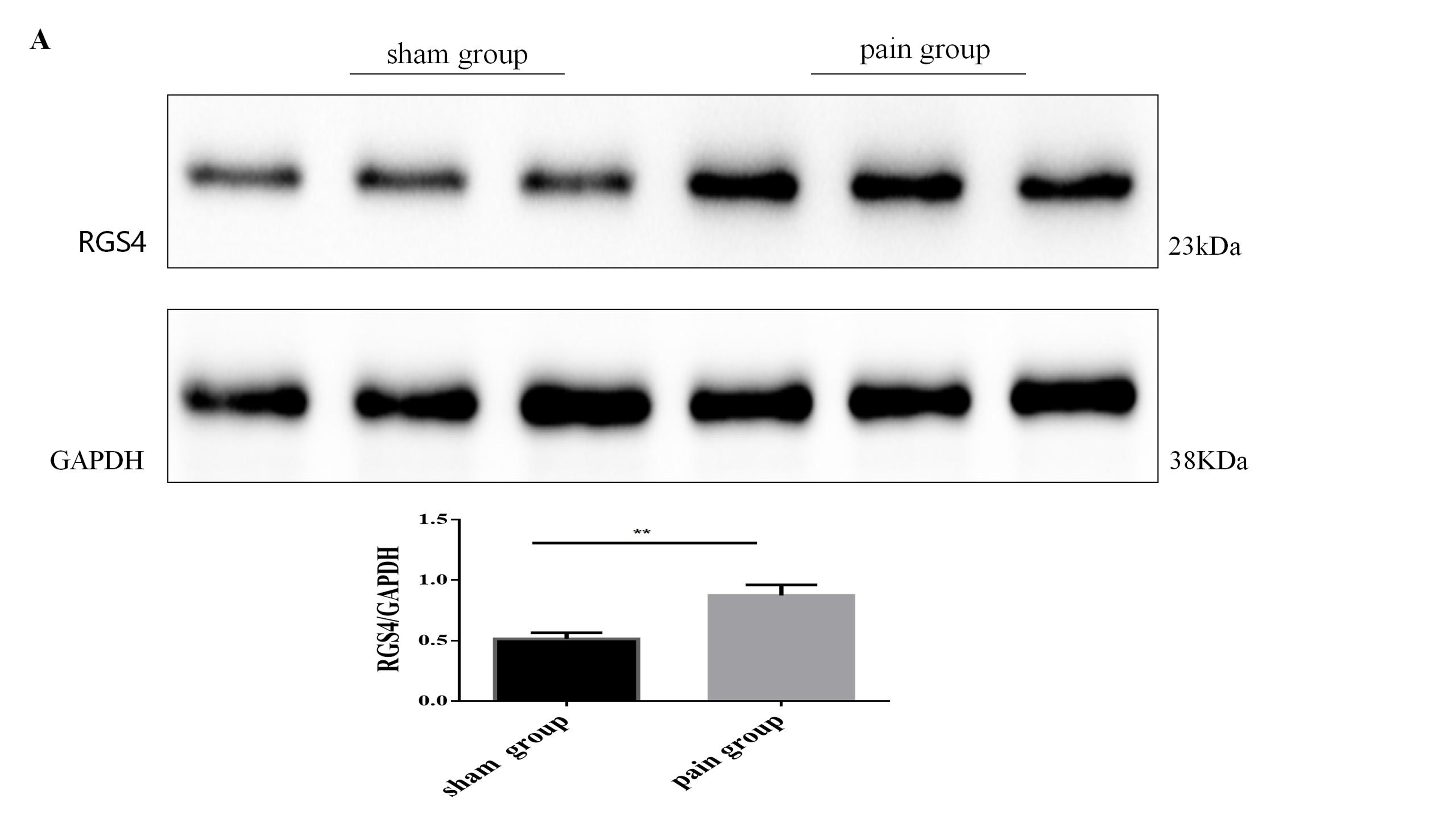

Supplement: Supplementary Figure S4 — The RGS4 protein expression by Western blot in sham and pain groups. The results are expressed as mean ± standard deviation. The histograms represent values. **represents p < 0.005 (two-tailed Student's t-tests). [file Image_4.JPEG]
